# Apoptosis in platelets is independent of the actin cytoskeleton

**DOI:** 10.1371/journal.pone.0276584

**Published:** 2022-11-15

**Authors:** Enoli De Silva, Manoj Paul, Hugh Kim

**Affiliations:** 1 Centre for Blood Research, University of British Columbia, Vancouver, British Columbia, Canada; 2 Department of Biochemistry and Molecular Biology, University of British Columbia, Vancouver, British Columbia, Canada; 3 Department of Oral Biological and Medical Sciences, University of British Columbia, Vancouver, British Columbia, Canada; National Institute of technology Rourkela, INDIA

## Abstract

Homeostasis between platelet production and clearance is essential for human health. A critical facet of the balance that facilitates platelet clearance from the circulation is apoptosis (programmed cell death). The precise cellular mechanisms that underpin platelet apoptosis are not defined. In nucleated cells, reorganization of the actin cytoskeleton is known to regulate platelet apoptosis. However, the role of the actin cytoskeleton in regulating apoptosis in platelets has not been extensively studied as they are anucleate and exhibit a distinctive physiology. Here, apoptosis was induced in washed human platelets using ABT-737, a BH3-mimetic drug. Mitochondrial depolarization was measured using the ratiometric dye JC-1; surface phosphatidylserine (PS) exposure was measured by annexin V binding; caspase-3 activation was measured by Western blotting. All three apoptotic markers were unaffected by the presence of either the actin depolymerizing drug cytochalasin D or the actin polymerizing drug jasplakinolide. Moreover, platelets were isolated from wild-type (*WT*) mice and mice deficient in gelsolin (*Gsn*), an actin-binding protein that is essential for normal cytoskeletal remodeling. In response to ABT-737, gelsolin-null (*Gsn*^*-/-*^) platelets initially showed accelerated PS exposure relative to *WT* platelets, however, both *WT* and *Gsn*^*-/-*^ platelets exhibited similar levels of mitochondrial depolarization and caspase-3 activation in response to ABT-737. We conclude that ABT-737 induces established markers of platelet apoptosis in an actin-independent manner.

## Introduction

Thrombocytopenia is defined by low numbers of circulating platelets (<150,000 platelets/μl of blood) that can lead to spontaneous bleeding. Low platelet counts may result from either deficient platelet production and/or overexuberant platelet clearance. Apoptosis, or programmed cell death, is a fundamental process by which cell populations are maintained. Published evidence from the past 15 years indicates that platelets also undergo apoptosis [1, 2]. Because they are anucleate and exhibit unique physiology compared to more comprehensively studied nucleated cells, the underlying molecular mechanisms of apoptosis remain poorly understood in platelets. However, the regulation of platelet apoptosis has clear implications for human health. For example, idiopathic thrombocytopenic purpura (ITP) is often a life-threatening condition that has been associated with increased apoptosis of platelets and/or megakaryocytes [3–5]. A clear comprehension of how apoptosis is regulated in platelets is therefore essential for the development of improved therapeutic approaches for managing thrombocytopenia.

Apoptosis in platelets typically follows the “intrinsic” pathway, which is initiated in a receptor-independent manner by stimulation that may be drug-induced [1, 6]. During intrinsic apoptosis, pro-apoptotic proteins including Bak and Bax translocate to the mitochondria and create pores that lead to the loss of mitochondrial membrane potential [7, 8]. The subsequent release of cytochrome c from the mitochondria leads to the cleavage, and activation, of the enzymes caspase-9 and caspase-3, culminating in cell death [9]. Notably, apoptotic platelets are also characterized by the exposure of phosphatidylserine (PS) on the plasma membrane and by morphological features including membrane blebbing. The pro-apoptotic function of Bak and Bax is constrained by the anti-apoptotic Bcl-2 proteins. Another group of proteins, termed BH3-only proteins, trigger apoptosis by dissociating Bcl-2 from Bak/Bax, thus nullifying the anti-apoptotic role of Bcl-2 [10]. Accordingly, BH3-mimetic drugs, including ABT-737 are widely used for the experimental induction of apoptosis [11]. The exact molecular mechanisms that transition platelets into an apoptotic phenotype remain incompletely understood.

The actin cytoskeleton is responsible for maintaining cellular structure integrity and has been implicated in the initiation and execution of apoptosis in nucleated cells [12]. Cytoskeletal rearrangements in response to various stimuli are due to a dynamic state of flux between globular G-actin monomers and filamentous F-actin polymers, which are characterized by a “barbed” end and “pointed” end. In response to stimuli, actin polymerization occurs as a result of monomer binding to the barbed end [13]. In nucleated cells, actin dynamics are involved in apoptosis by participating in the accumulation of reactive oxygen species (ROS) [14], mitochondrial depolarization [15], caspase activation [16], cytochrome c release [17], and apoptotic body formation [12, 18]. Furthermore, actin reportedly regulates the activation of Bcl-2 proteins [19, 20]. Apoptotic morphological changes depend heavily on a dynamic actin cytoskeleton [21, 22] and the actin-binding proteins that regulate these processes [23].

Gelsolin is an ~90-kDa actin-binding protein with six homology domains (G1-G6) that respond to specific cellular conditions by regulating actin dynamics [24, 25]. Upon binding calcium or under low pH conditions in the absence of calcium, gelsolin binds and severs actin filaments, then caps the barbed ends of the newly formed actin fragments, thereby promoting actin depolymerisation [25, 26]. Conversely, phosphatidylinositol lipids (PIP_2_) bind and release gelsolin from actin, promoting actin polymerization [25]. In addition to the importance of gelsolin in actin dynamics, gelsolin can play a major role in promoting or inhibiting apoptosis. The caspase-3-cleaved N-terminal gelsolin fragment was found to induce apoptosis in activated hepatic stellate cells [27]. In contrast, gelsolin overexpression reportedly protects nucleated cells from apoptosis [28, 29]. In human platelets, apoptosis induced by the BH3-mimetic drug, ABT-737, leads to gelsolin cleavage [30]. These studies highlight a potential role for gelsolin and the actin cytoskeleton in platelet apoptosis; however, the involvement of the actin cytoskeleton in mediating platelet apoptosis has not been studied to date.

We hypothesized that the actin cytoskeleton is a critical determinant for pro-apoptotic platelet induction, consistent with observations in nucleated cells. We initially sought to confirm the role of the actin cytoskeleton in platelet apoptosis by analyzing well-established apoptotic markers in the presence of cytoskeleton-modulating drugs. As expected, ABT-737 induced mitochondrial depolarization, membrane surface phosphatidylserine (PS) exposure and caspase-3 cleavage in human platelets. However, these endpoints were surprisingly unaffected by the disruption of the actin cytoskeleton by cytochalasin D; they were also unaffected by the induction of actin polymerization by jasplakinolide. Furthermore, we studied the apoptotic response to ABT-737 in wild-type (*WT*) and gelsolin-null (*Gsn*^*-/-*^) mouse platelets. Again, we found that mitochondrial depolarization, PS exposure and caspase-3 cleavage were largely unaffected by the loss of gelsolin expression in platelets. Our findings indicate that ABT-737-mediated apoptosis in platelets occurs independently of the actin cytoskeleton.

## Materials and methods

### Reagents

Prostaglandin E1 (PGE1), heparin, ABT-737, cytochalasin D and jasplakinolide were purchased from Millipore-Sigma (Oakville, ON, Canada). The JC-1 ratiometric dye and the Alexa-Fluor-488-conjugated Annexin V dye were purchased from Life Technologies (Grand Island, NY). The anti-caspase-3 antibody (catalogue #: 9665P), the anti-cleaved caspase-3 antibody (catalogue #: 9664), the anti-gelsolin antibody (catalogue #: 12953) and the HRP-conjugated anti-beta-tubulin antibody (catalogue #: 5346) were purchased from Cell Signaling Technologies (Danvers, MA). The HRP-conjugated anti-beta-actin antibody (catalogue #: SC-47778 HRP) was purchased from Santa Cruz.

### Human platelet preparation

Platelets were collected from healthy volunteers, with informed consent and approval from the University of British Columbia Clinical Research Ethics Board (CREB) in accordance with the Declaration of Helsinki. Each experimental data set was derived from blood obtained from three different donors. Blood was collected in tubes containing acid-citrate-dextrose (ACD) buffer, and platelet-rich plasma (PRP) was separated by centrifugation for 10 minutes at 200*g*. Platelets were isolated by centrifugation for 10 minutes at 800*g* in the presence of 1 μM prostaglandin E1. Centrifugation was performed at a slow rate of deceleration to prevent inadvertent platelet activation. The platelets were washed in the presence of 3.5 mg/mL of bovine serum albumin (BSA), 10 U heparin, and 1 μM PGE1 for 10 minutes at 37°C and then centrifuged for 10 minutes at 800*g*. The washed platelets were resuspended in Tyrodes buffer (0.37 mM NaH_2_PO_4_, 2.7 mM KCl, 137 mM NaCl, 10 mM HEPES, 11.9 mM NaHCO_3_, 1 mM MgCl_2_, 5.5 mM glucose, pH = 7.4) and allowed to rest for 30 minutes at 37°C before using for experiments.

### Mice

Gelsolin heterozygous (*Gsn*^*+/-*^) mice on an FVB background [31] were kindly donated by Gavin Oudit (University of Alberta) and Chris McCulloch (University of Toronto). Gelsolin-null (*Gsn*^*-/-*^) mice and littermate wild-type controls were generated by crossing the heterozygous mice and then identified by PCR genotyping. Each experimental data set used platelets derived from blood obtained from 3 wild-type and 3 knockout male mice (aged 8–12 weeks).

### Mouse platelet preparation

Animal work was conducted with approval from the University of British Columbia’s Animal Care Committee (ACC, protocol #A21-0177). Mouse blood was collected via retro-orbital plexus bleeding into tubes containing acid-citrate-dextrose (ACD) and 2 U heparin. Mice were sacrificed by cervical dislocation. The blood was carefully transferred to 5 mL Eppendorf tubes with 2 mL Tyrode’s buffer (0.37 mM NaH_2_PO_4_, 2.7 mM KCl, 137 mM NaCl, 10 mM HEPES, 11.9 mM NaHCO_3_, 1 mM MgCl_2_, 5.5 mM glucose, pH = 7.4), and PRP was isolated after centrifugation for 7 minutes at 200*g*. Platelets were isolated from the PRP after centrifugation for 10 minutes at 800*g*, and resuspended in Tyrode’s buffer.

### Induction of apoptosis and measurement of apoptotic endpoints

Washed human platelets (2x10^8^ cells/mL) were pre-treated with 10 μM cytochalasin D or 1 μM jasplakinolide for 40 minutes at room temperature (RT), followed by treatment with either 1 or 10 μM ABT-737 or vehicle for the specified times at 37°C. Mouse platelets were directly stimulated with either 1 or 10 μM ABT-737 or vehicle for the specified times. Mitochondrial depolarization was measured by flow cytometry after incubating the stimulated platelets in 2 μg/mL JC-1 ratiometric dye for 20 minutes at 37°C. PS exposure was measured by flow cytometry after incubating the stimulated platelets in Alexa-Fluor-488-conjugated Annexin V dye in the presence of 2.5 mM calcium for 20 minutes at RT. The concentration of the Annexin V dye was used according to the manufacturer’s instructions.

### SDS-PAGE and immunoblotting

Washed platelets (5x10^8^ cells/mL) were lysed in 5X NP-40 lysis buffer (250 mM Tris, 750 mM NaCl, 5% (v/v) NP-40, pH = 7.4) and 5 mM EDTA, and resuspended in Laemelli’s sample buffer. Samples were boiled at 95°C for 5 minutes prior to loading on a Tris-glycine gel, then transferred onto a PVDF membrane and blocked in 3% (w/v) BSA solution in TBS-T. Western blot analysis was performed using rabbit anti-cleaved-caspase-3 and rabbit anti-caspase-3 antibodies, and detected using enhanced chemiluminescence (ECL).

### Statistical analysis

All statistical evaluations were performed using GraphPad Prism 9 software. Differences between groups were analyzed using either Student’s t-test or a two-way analysis of variance (ANOVA) and Bonferroni post-hoc multiple comparison tests, as appropriate. Statistical significance was set at p<0.05.

## Results

### Mitochondrial depolarization, PS exposure and caspase-3 cleavage in platelets are not affected by disassembly of the actin cytoskeleton

**Fig 1:**
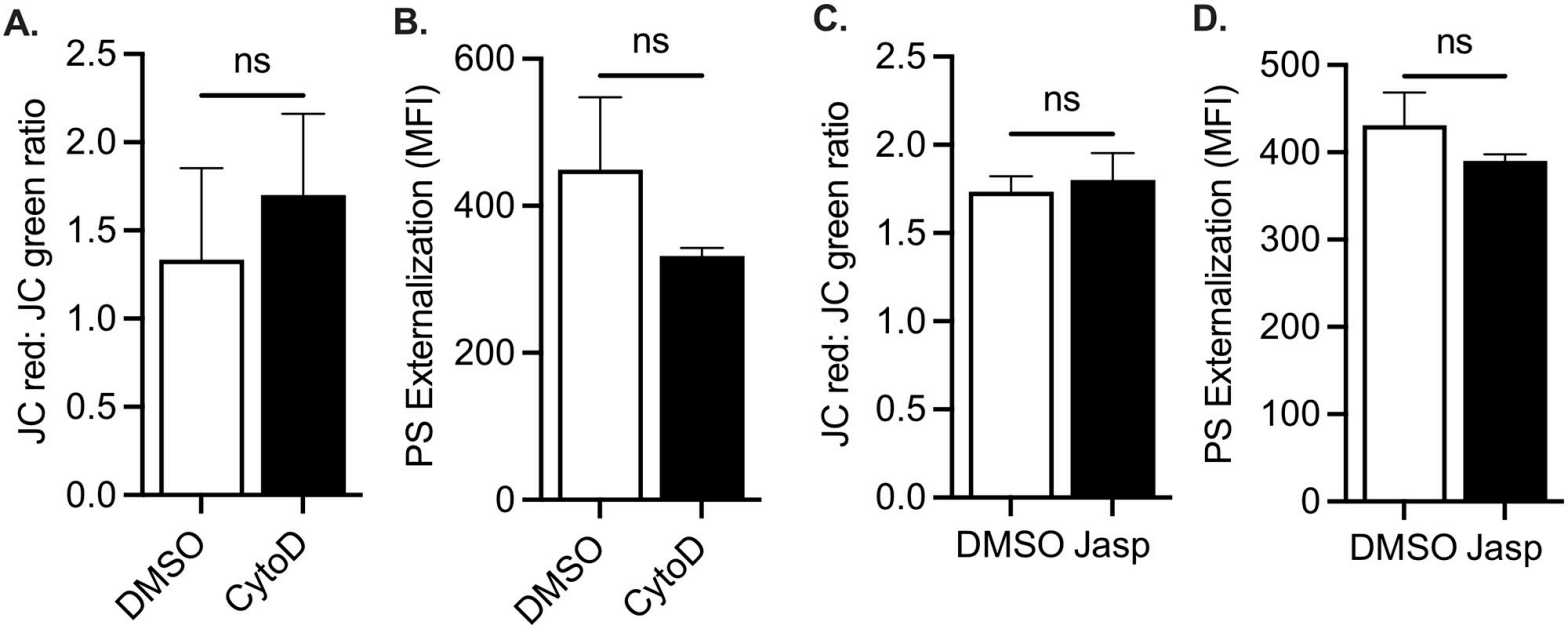
Mitochondrial depolarization and PS externalization in human platelets are not affected by actin depolymerization and polymerization. **A**. Mitochondrial depolarization was measured in washed human platelets using the JC-1 dye. Bar graph depicts the loss of mitochondrial membrane potential in platelets treated with DMSO vehicle alone (DMSO, white bars) or with 10 μM cytochalasin D alone (CytoD, black bars). Data are mean ± SEM, analyzed by t-test, and represent a minimum of 3 independent experiments using blood from different donors. **B**. Phosphatidylserine (PS) exposure was measured in washed platelets using the Annexin V dye. Bar graph depicts PS exposure in platelets treated with DMSO vehicle alone (DMSO, white bars) or with 10 μM cytochalasin D alone (CytoD, black bars). Data are mean ± SEM, analyzed by t-test, and represent a minimum of 3 independent experiments using blood from different donors. **C**. Mitochondrial depolarization was measured in washed human platelets using the JC-1 dye. Bar graph depicts the loss of mitochondrial membrane potential in platelets treated with DMSO vehicle alone (DMSO, white bars) or with 1 μM jasplakinolide alone (Jasp, black bars). Data are mean ± SEM, analyzed t-test, and represent a minimum of 3 independent experiments using blood from different donors. **D**. Phosphatidylserine (PS) exposure was measured in washed platelets using the Annexin V dye. Bar graph depicts PS exposure in platelets treated with DMSO vehicle alone (DMSO, white bars) or with 1 μM jasplakinolide alone (Jasp, black bars). Data are mean ± SEM, analyzed by using t-test, and represent a minimum of 3 independent experiments using blood from different donors.

**Fig 2:**
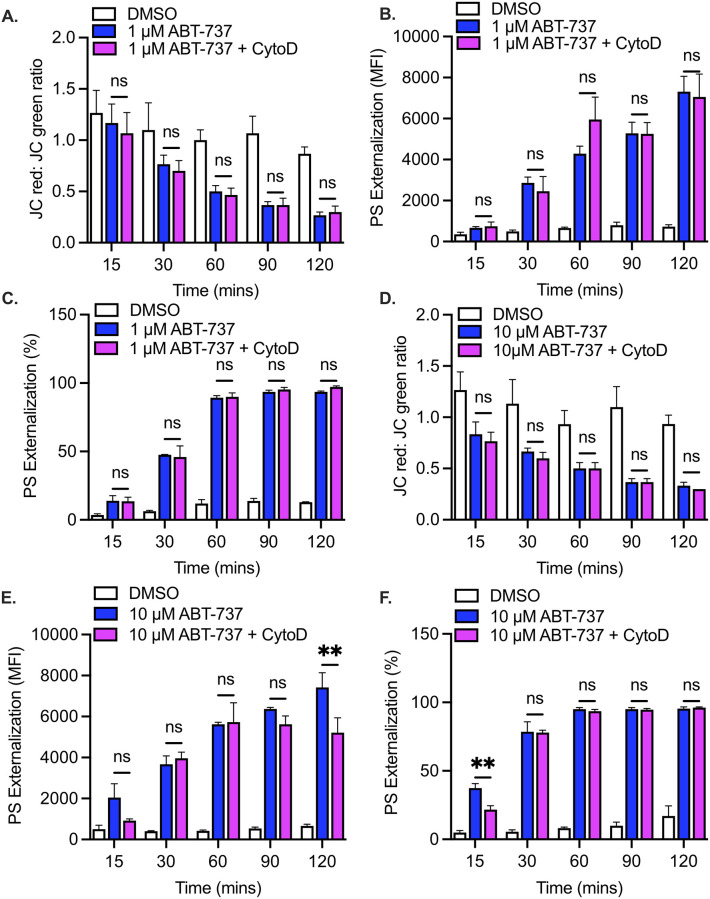
ABT-737-induced mitochondrial depolarization and PS externalization in human platelets are not affected by actin depolymerization. **A**. Mitochondrial depolarization was measured in washed human platelets using the JC-1 dye. Bar graph depicts the loss of mitochondrial membrane potential in platelets treated with DMSO vehicle alone (DMSO, white bars), with 1 μM ABT-737 (ABT-737, blue bars) or with 1 μM ABT plus the actin depolymerizing agent cytochalasin D (ABT-737+CytoD, pink bars). Data are mean ± SEM, analyzed by 2-way ANOVA and Bonferroni post-hoc multiple comparison tests, and represent a minimum of 3 independent experiments using blood from different donors. **B, C**. Phosphatidylserine (PS) exposure was measured in washed platelets using the Annexin V dye. Bar graphs depict PS exposure in platelets treated with DMSO vehicle alone (DMSO, white bars), with 1 μM ABT-737 (ABT-737, blue bars) or with 1 μM ABT plus the actin depolymerizing agent cytochalasin D (ABT-737+CytoD, pink bars). Data are expressed both in terms of mean fluorescence intensity **(B)** and as a percentage (%) of PS-positive platelets **(C)**. Data are mean ± SEM, analyzed by using 2-way ANOVA and Bonferroni post-hoc multiple comparison tests, and represent a minimum of 3 independent experiments using blood from different donors. **D**. Bar graph depicts the loss of mitochondrial membrane potential in platelets treated with DMSO vehicle alone (DMSO, white bars), with 10 μM ABT-737 (ABT-737, blue bars) or with 10 μM ABT plus the actin depolymerizing agent cytochalasin D (ABT-737+CytoD, pink bars). Data are mean ± SEM, analyzed 2-way ANOVA and Bonferroni post-hoc multiple comparison tests and represent a minimum of 3 independent experiments using blood from different donors. **E, F**. Bar graphs depict PS exposure in platelets treated with DMSO vehicle alone (DMSO, white bars), with 10 μM ABT-737 (ABT-737, blue bars) or with 10 μM ABT plus the actin depolymerizing agent cytochalasin D (ABT-737+CytoD, pink bars). Data are expressed both in terms of mean fluorescence intensity **(E)** and as a percentage (%) of PS-positive platelets **(F)**. Data are mean ± SEM, analyzed by 2-way ANOVA and Bonferroni post-hoc multiple comparison tests, and represent a minimum of 3 independent experiments using blood from different donors.

To test the hypothesis that pro-apoptotic signaling is contingent on the integrity of the actin cytoskeleton, we first pre-treated platelets with the actin depolymerizing drug cytochalasin D, which binds to the fast-growing plus (“barbed”) ends of actin filaments and prevents further polymerization [32]. In addition, given the bi-directional nature of actin dynamics (assembly and disassembly), we also probed the role of the actin cytoskeleton in apoptosis using jasplakinolide, an agonist that induces actin polymerization by stabilizing the actin barbed ends [33]. Treatment of human platelets with cytochalasin D or jasplakinolide alone did not in and by themselves induce mitochondrial depolarization and PS exposure (Fig 1A–1D). As expected, mitochondrial depolarization, measured by the ratiometric JC-1 dye, occurred in platelets following treatment with the BH3-mimetic ABT-737 (1 μM) (Fig 2A). Surprisingly, a similar response (p>0.05) was observed both in the presence or absence of cytochalasin D (Fig 2A). Similarly, ABT-737 induced an increase in phosphatidylserine (PS) exposure in platelets; this response was unaffected (p>0.05) by cytochalasin D (Fig 2B and 2C). To verify that the PS expression is representative of the entire platelet population, we analyzed and expressed the PS exposure data in terms of overall mean fluorescence intensity (MFI) (Fig 2B) and as a function of the percentage (%) of PS-exposing platelets (Fig 2C). Both methods of analysis indicated that cytochalasin D treatment did not affect ABT-737-induced PS exposure.

Similar results were obtained when platelet apoptosis was induced with a higher concentration (10 μM) of ABT-737 (Fig 2D–2F). Cytochalasin D reduced PS exposure (MFI) at the 120-minute time point (Fig 2E), however, the corresponding percentages of PS-exposing platelets were similar (Fig 2F). Cytochalasin D also reduced the % of PS-exposing platelets at 15 minutes (Fig 2F) but the corresponding difference in MFI was not significant (Fig 2E).

**Fig 3:**
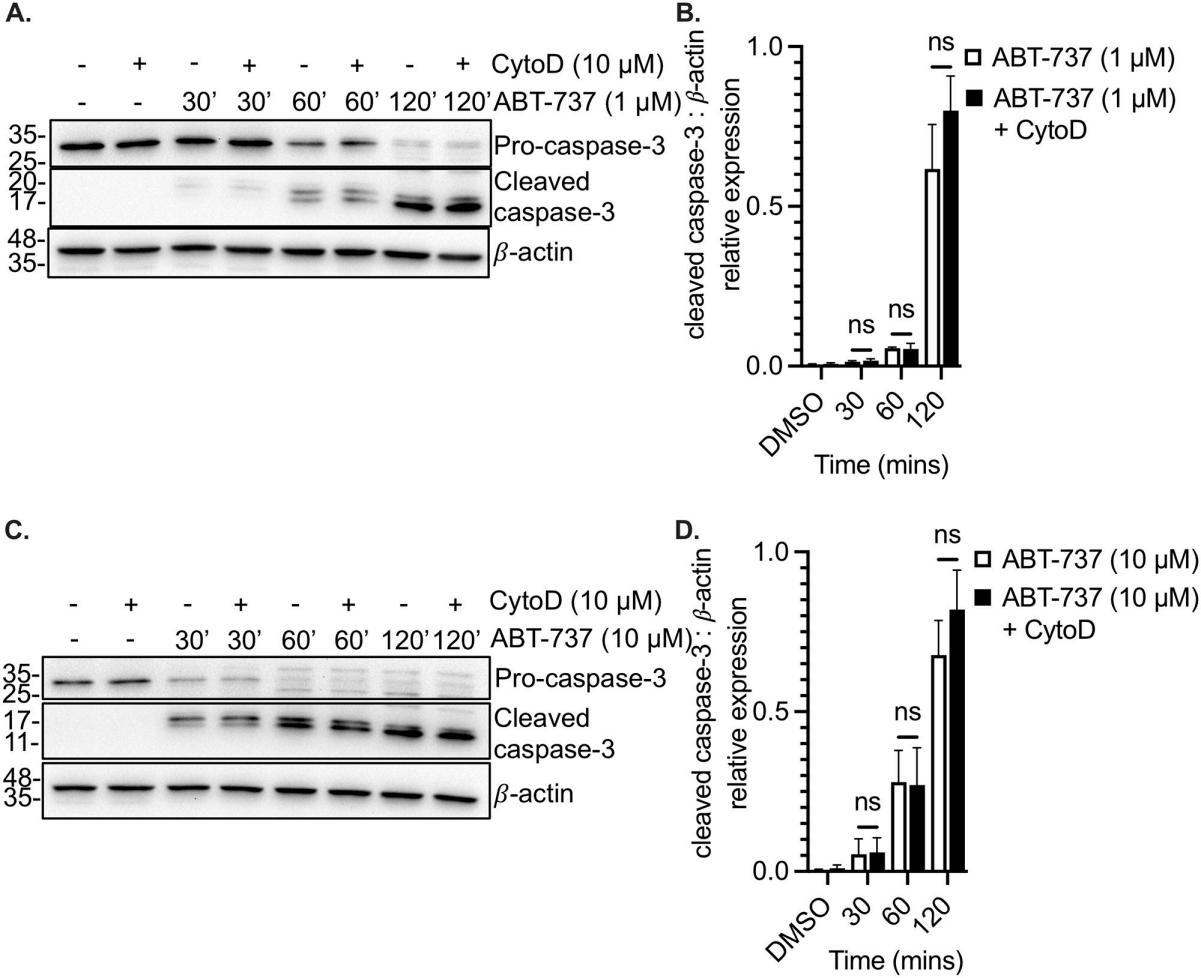
ABT-737-induced caspase-3 cleavage in human platelets is not affected by actin depolymerization. **A**. Washed human platelets were incubated in the absence (-) or presence (+) of the actin depolymerizing drug cytochalasin D, and in the absence or presence of 1 μM ABT-737, for the indicated times. Equal amounts of platelet lysate were resolved by SDS-PAGE; immunoblots were probed for pro-caspase-3 and cleaved caspase-3. Beta-actin (β-actin) is shown as a loading control. **B**. Bar graph depicts caspase-3 cleavage (normalized to beta-actin levels) induced by 1 μM ABT-737 in the absence (ABT-737, white bars) or presence (ABT-737+CytoD) of cytochalasin D. Data are mean ± SEM, analyzed by 2-way ANOVA and Bonferroni post-hoc multiple comparison tests, and represent a minimum of 3 independent experiments using blood from different donors. **C**. Immunoblots represent pro-caspase-3 and cleaved caspase-3 levels in platelets treated with 10 μM ABT-737, in the presence or absence of cytochalasin D. Beta-actin (β-actin) is shown as a loading control. **D**. Bar graph depicts caspase-3 cleavage (normalized to beta-actin levels) induced by 10 μM ABT-737 in the absence (ABT-737, white bars) or presence (ABT-737+CytoD) of cytochalasin D. Data are mean ± SEM, analyzed by 2-way ANOVA and Bonferroni post-hoc multiple comparison tests, and represent a minimum of 3 independent experiments using blood from different donors.

The cleavage of caspase-3 is the terminal step in the intrinsic apoptotic pathway, and this was observed in human platelets treated with ABT-737 for 30–120 minutes (Fig 3A). As was observed with mitochondrial depolarization and PS exposure, the degree of caspase-3 cleavage was unaffected by cytochalasin D (Fig 3A and 3B). While the higher concentration (10 μM) of ABT-737 accelerated the cleavage of caspase-3, similar results were observed in the presence and absence of cytochalasin D (Fig 3C and 3D). Taken together, these data indicate that the intrinsic pathway of platelet apoptosis proceeds independently of an intact actin cytoskeleton.

### Induction of actin polymerization by jasplakinolide does not affect mitochondrial depolarization, PS exposure or caspase-3 cleavage

**Fig 4:**
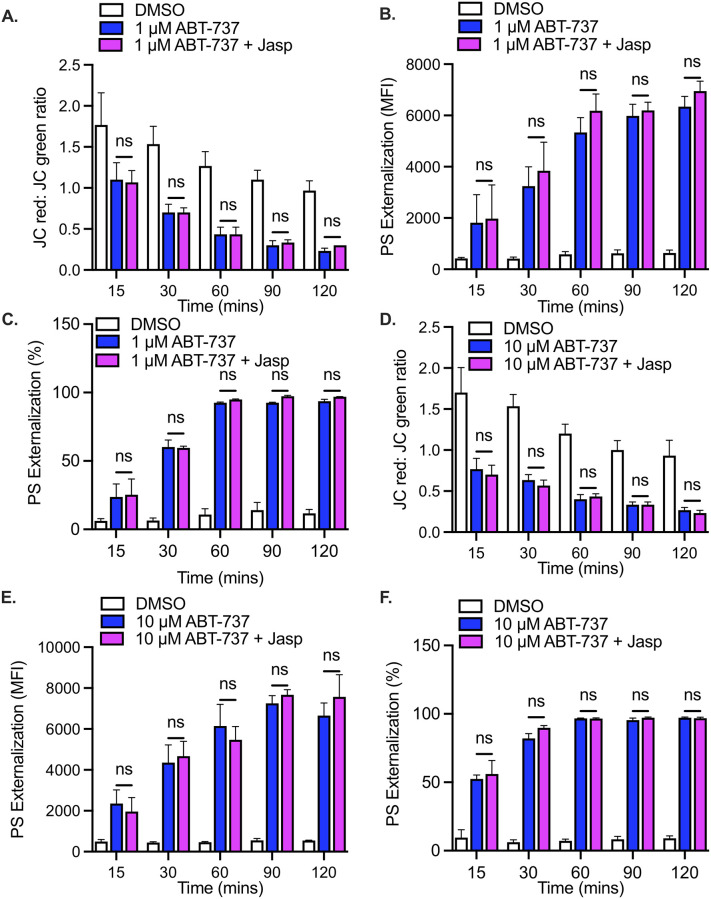
ABT-737-induced mitochondrial depolarization and PS externalization in human platelets are not affected by the induction of actin polymerization. **A**. Mitochondrial depolarization was measured in washed human platelets using the JC-1 dye. Bar graph depicts the loss of mitochondrial membrane potential in platelets treated with DMSO vehicle alone (DMSO, white bars), with 1 μM ABT-737 (ABT-737, blue bars) or with 1 μM ABT plus the actin polymerizing agent jasplakinolide (ABT-737+Jasp, pink bars). Data are mean ± SEM, analyzed by 2-way ANOVA and Bonferroni post-hoc multiple comparison tests, and represent a minimum of 3 independent experiments using blood from different donors. **B, C**. Phosphatidylserine (PS) exposure was measured in washed platelets using the Annexin V dye. Bar graphs depict PS exposure in platelets treated with DMSO vehicle alone (DMSO, white bars), with 1 μM ABT-737 (ABT-737, blue bars) or with 1 μM ABT plus the actin polymerizing agent jasplakinolide (ABT-737+Jasp, pink bars). Data are expressed both in terms of mean fluorescence intensity **(B)** and as a percentage (%) of PS-positive platelets **(C)**. Data are mean ± SEM, analyzed by using 2-way ANOVA and Bonferroni post-hoc multiple comparison tests, and represent a minimum of 3 independent experiments using blood from different donors. **D**. Bar graph depicts the loss of mitochondrial membrane potential in platelets treated with DMSO vehicle alone (DMSO, white bars), with 10 μM ABT-737 (ABT-737, blue bars) or with 10 μM ABT plus the actin polymerizing agent jasplakinolide (ABT-737+Jasp, pink bars). Data are mean ± SEM, analyzed 2-way ANOVA and Bonferroni post-hoc multiple comparison tests and represent a minimum of 3 independent experiments using blood from different donors. **E, F**. Bar graphs depict PS exposure in platelets treated with DMSO vehicle alone (DMSO, white bars), with 10 μM ABT-737 (ABT-737, blue bars) or with 10 μM ABT plus the actin polymerizing agent jasplakinolide (ABT-737+Jasp, pink bars). Data are expressed both in terms of mean fluorescence intensity **(E)** and as a percentage (%) of PS-positive platelets **(F)**. Data are mean ± SEM, analyzed by 2-way ANOVA and Bonferroni post-hoc multiple comparison tests, and represent a minimum of 3 independent experiments using blood from different donors.

**Fig 5:**
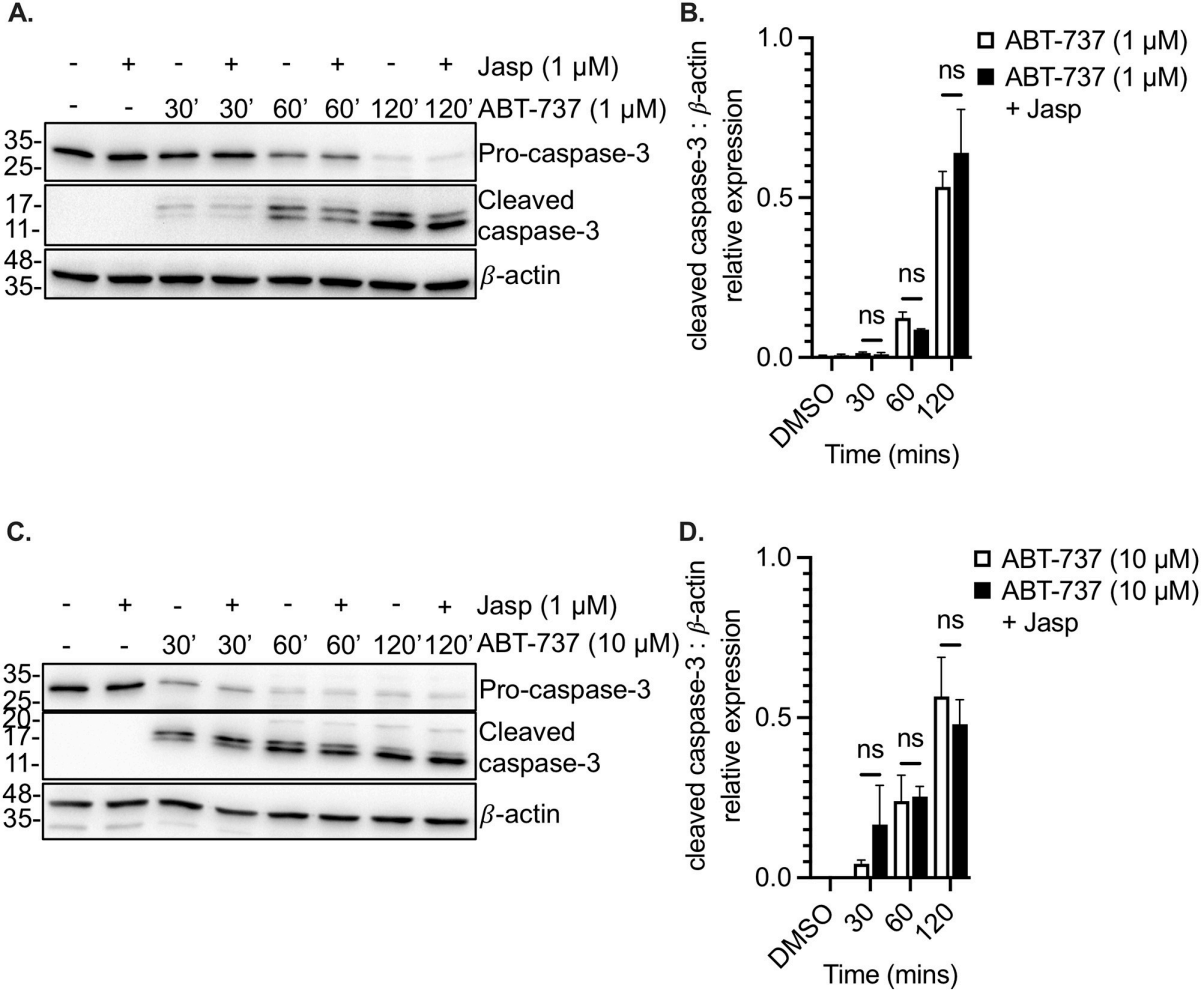
ABT-737-induced caspase-3 cleavage in human platelets is not affected by the induction of actin polymerization. **A**. Washed human platelets were incubated in the absence (-) or presence (+) of the actin polymerizing drug jasplakinolide, and in the absence or presence of 1 μM ABT-737, for the indicated times. Equal amounts of platelet lysate were resolved by SDS-PAGE; immunoblots were probed for pro-caspase-3 and cleaved caspase-3. Beta-actin (β-actin) is shown as a loading control. **B**. Bar graph depicts caspase-3 cleavage (normalized to beta-actin levels) induced by 1 μM ABT-737 in the absence (ABT-737, white bars) or presence (ABT-737+Jasp) of jasplakinolide. Data are mean ± SEM, analyzed by 2-way ANOVA and Bonferroni post-hoc multiple comparison tests, and represent a minimum of 3 independent experiments using blood from different donors. **C**. Immunoblots represent pro-caspase-3 and cleaved caspase-3 levels in platelets treated with 10 μM ABT-737, in the presence or absence of jasplakinolide. Beta-actin (β-actin) is shown as a loading control. **D**. Bar graph depicts caspase-3 cleavage (normalized to beta-actin levels) induced by 10 μM ABT-737 in the absence (ABT-737, white bars) or presence (ABT-737+Jasp) of jasplakinolide. Data are mean ± SEM, analyzed by 2-way ANOVA and Bonferroni post-hoc multiple comparison tests, and represent a minimum of 3 independent experiments using blood from different donors.

As was observed in platelets treated with CytoD, the mitochondrial depolarization and surface PS exposure induced by ABT-737 was not significantly (p>0.05) affected by pre-treatment with jasplakinolide (Fig 4A–4C). Similar results were obtained with the higher dose of ABT-737 (Fig 4D–4F). Furthermore, caspase-3 cleavage was unaffected by jasplakinolide, at both the low and high concentrations of ABT-737 (Fig 5A–5D).

### Platelet apoptosis is unaffected by the loss of gelsolin expression

**Fig 6:**
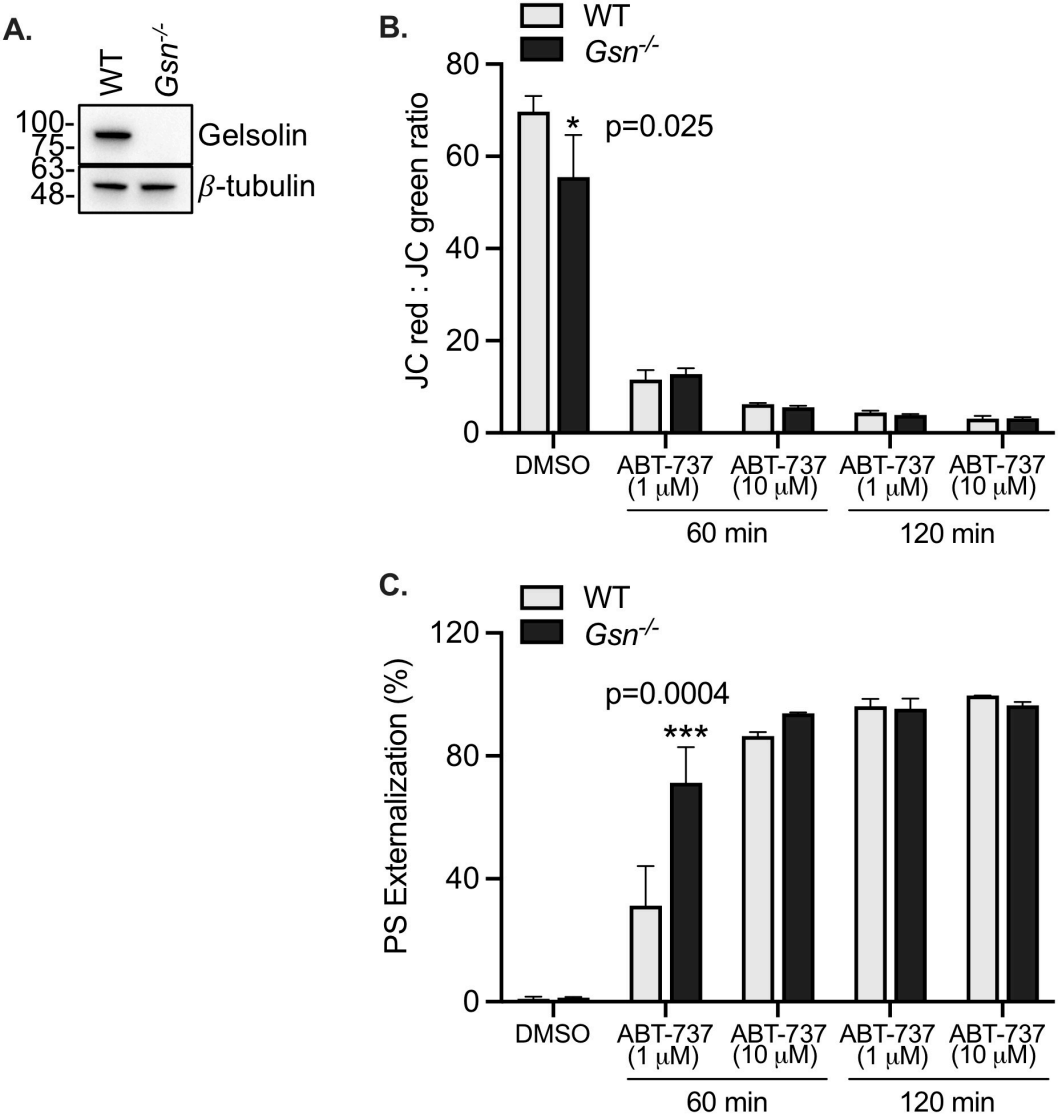
ABT-737-induced mitochondrial depolarization and PS externalization in mouse platelets is unchanged by the loss of gelsolin. **A**. Western blot confirms the deletion of gelsolin expression in mouse platelets (*WT*, wild-type; *Gsn*^*-/-*^, knockout). β-tubulin is shown as a loading control. **B**. Mitochondrial depolarization was measured in washed mouse wild-type (*WT*, grey bars) and gelsolin-null (*Gsn*^*-/-*^, black bars) platelets using the JC-1 dye. Bar graph depicts the loss of mitochondrial membrane potential in platelets treated with DMSO vehicle alone, with 1 μM ABT-737 or with 10 μM ABT, for 60 or 120 minutes as indicated. Data are mean ± SEM, analyzed by 2-way ANOVA and Bonferroni post-hoc multiple comparison tests, and represent a minimum of 3 independent experiments. **C**. Phosphatidylserine (PS) exposure was measured in washed mouse wild-type (*WT*, grey bars) and gelsolin-null (*Gsn*^*-/-*^, black bars) platelets using the Annexin V dye. Bar graphs depict PS exposure in platelets treated with DMSO vehicle alone, with 1 μM ABT-737 or with 10 μM ABT, for 60 or 120 minutes as indicated. Data are mean ± SEM, analyzed by using 2-way ANOVA and Bonferroni post-hoc multiple comparison tests, and represent a minimum of 3 independent experiments.

**Fig 7:**
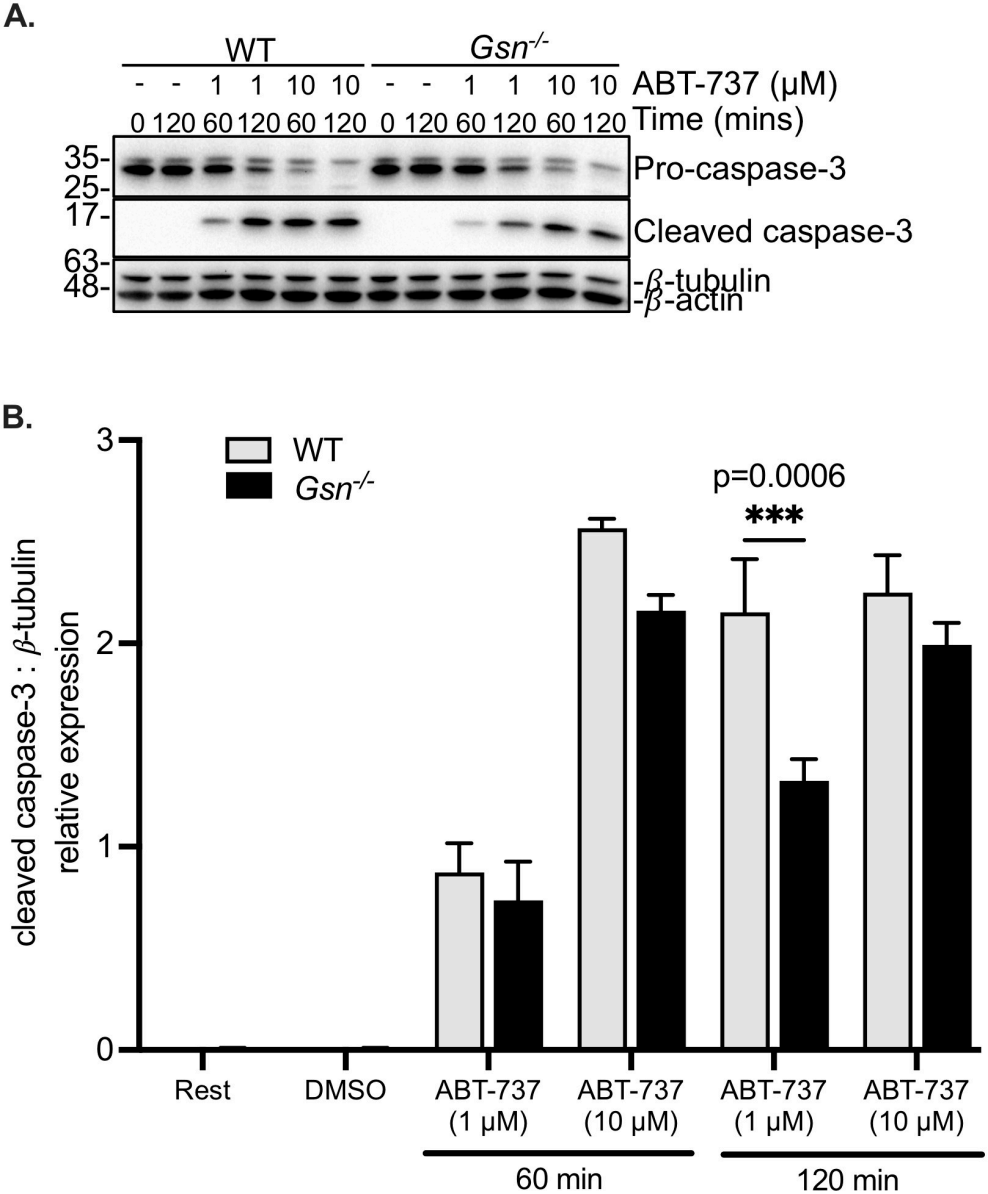
ABT-737-induced caspase-3 cleavage in mouse platelets is unchanged by the loss of gelsolin. **A**. Washed wild-type (*WT*) and gelsolin-null (*Gsn*^*-/-*^) platelets were treated with DMSO vehicle alone, with 1 μM ABT-737 or with 10 μM ABT, for 60 or 120 minutes as indicated. Equal amounts of lysate were resolved by SDS-PAGE; immunoblots were probed for pro-caspase-3 and cleaved caspase-3. Beta-actin (β-actin) and beta-tubulin (β-tubulin) are shown as loading controls. **B**. Bar graph depicts caspase-3 cleavage (normalized to beta-tubulin levels) in wild-type (*WT*, grey bars) and gelsolin-null (*Gsn*^*-/-*^, black bars) platelets were treated with DMSO vehicle alone, with 1 μM ABT-737 or with 10 μM ABT, and for 60 or 120 minutes as indicated. Data are mean ± SEM, analyzed by 2-way ANOVA and Bonferroni post-hoc multiple comparison tests, and represent a minimum of 3 independent experiments.

To further verify the independence of platelet apoptosis from the actin cytoskeleton, we used mice deficient in gelsolin, a protein that modulates the cytoskeleton by severing actin filaments thus ensuring a supply of free barbed ends [25], and which is also cleaved during platelet apoptosis [30, 34]. Deletion of gelsolin expression in the knockout (*Gsn*^*-/-*^) mice was verified by immunoblotting (Fig 6A). Treatment of the mouse platelets with ABT-737 induced mitochondrial depolarization and increased surface PS exposure, at both low (1 μM) and high (10 μM) concentrations of ABT-737 and at both time points observed (Fig 6B and 6C). *WT* and *Gsn*^*-/-*^ platelets exhibited similar degrees of mitochondrial depolarization (Fig 6B) and surface PS exposure (Fig 6C) in response to ABT-737. It was noted that *Gsn*^*-/-*^ platelets displayed greater (p<0.05) surface PS exposure than the *WT* controls after 60 minutes of treatment with 1 μM ABT-737; however, this effect was not observed at the higher concentration of ABT-737 (Fig 6C). In addition, ABT-737 induced caspase-3 cleavage in mouse platelets (Fig 7A). Levels of cleaved caspase-3 were lower in *Gsn*^*-/-*^ platelets relative to controls although this was only statistically significant at 1 μM of ABT-737 at the 120-minute time point (Fig 7B). Collectively, the data obtained from human and mouse platelets strongly suggest that the intrinsic pathway of apoptosis is independent of the actin cytoskeleton.

## Discussion

Apoptosis is a physiologic process that determines platelet lifespan [2] although the exact molecular mechanisms underlying apoptosis are undefined. Dysregulation of platelet apoptosis has clear implications for human health. For example, immune thrombocytopenia (ITP) is an autoimmune condition characterized by antibodies targeting platelet antigens [35]. Auto-antibodies targeting the GPIIb-IIIa and GPIb-IX platelet receptors induce apoptotic signaling in platelets, contributing to ITP [3, 36]. Conversely, the induction of platelet apoptosis may have therapeutic benefits in the treatment of atherosclerosis [37]. Consequently, an improved understanding of the pro- and anti-apoptotic signaling mechanisms in platelets will aid in advancing improved therapeutic approaches for multiple diseases whose outcomes are influenced by circulating platelet availability. The actin cytoskeleton and associated actin-binding proteins are central to multiple cellular functions [38]. Previous studies on nucleated cells have highlighted the role of the actin cytoskeleton in regulating apoptosis [12, 14–19]. In the present study, we used both an inhibitor and activator of actin polymerization to address this question in detail. In addition, platelets from the *Gsn*^*-/-*^ mice provided an additional tool to explore the contribution of the cytoskeleton on platelet apoptosis. The overexpression of gelsolin in Jurkat cells was shown to inhibit mitochondrial depolarization and cytochrome c release under apoptotic conditions stimulated by Fas-antibody, staurosporine, thapsigargin and protoporphyrin IX [29]. By contrast, in our study, we found that ABT-737-induced mitochondrial depolarization in platelets was unaffected by the loss of gelsolin. Our data from the actin-modulating drugs and the *Gsn*^*-/-*^ platelets collectively indicate that ABT-737-induced platelet apoptosis is independent of the actin cytoskeleton.

Our findings underscore the unique nature of platelet structure and function. For example, the organelle composition in platelets is clearly distinct from that of nucleated cells. Conceivably, this fundamental difference would alter the nature of pro-apoptotic signaling in platelets. For example, the nucleus is a major intracellular target during apoptosis. Caspases degrade the nuclear envelope, allowing entry of caspase and nuclease, leading to chromatin condensation and DNA degradation [39, 40]. Notably, jasplakinolide reportedly activates DNase I in HL-60 cells [12, 41], suggesting that the actin cytoskeleton is involved in nuclear DNA degradation. The mitochondria is another prominent organelle targeted by Bax proteins to promote cytochrome c release during apoptosis [42]. Again, the actin cytoskeleton is likely involved in this process since latrunculin A treatment of MCF10A cells increased Bax translocation to the mitochondria [19]. Since the mitochondria is a gateway during the cell’s commitment to apoptosis, the cytoskeleton may therefore serve as an anti-apoptotic barrier. However, because mitochondria are markedly less abundant in platelets than in nucleated cells [43], it is plausible that cytoskeleton dynamics, with respect to mitochondrial depolarization, may be insignificant in the context of platelet apoptosis. The differences between platelets and nucleated cells are reflected in other studies that examined the role of gelsolin in apoptosis. For example, Koya *et al* reported that Jurkat cells overexpressing gelsolin showed reduced mitochondrial potential loss and caspase activation in response to apoptotic agents, suggesting an anti-apoptotic role for gelsolin [29]. In contrast, Kothakota *et al* reported that gelsolin expression in neutrophils had no effect on caspase-3 activation, but that a loss of gelsolin was associated with a delayed onset of apoptotic morphological changes in response to tumor necrosis factor (TNF) plus cycloheximide [34], suggesting a pro-apoptotic role for gelsolin. Collectively, findings from our group and others suggest that the role of gelsolin (and by extension, the actin cytoskeleton) in apoptosis may be highly cell type-specific.

There was general concordance among the outcomes of mitochondrial depolarization, caspase-3 cleavage, and PS exposure in that all 3 experimental endpoints in platelets were largely unaffected by cytoskeleton-modulating drugs or by the loss of gelsolin expression. Interestingly, we observed that disruption of the cytoskeleton appeared to exert a different effect on PS exposure, at certain time points. We noted that ABT-737-induced PS exposure was reduced by cytochalasin D treatment at both the early (15 minute) and later (120 minute) time points after ABT-737 treatment. Conversely, the loss of gelsolin accelerated ABT-737-induced PS exposure. In both cases, these findings were not mirrored by changes in mitochondrial depolarization or caspase-3 cleavage; we instead noted a trend of reduced caspase-3 cleavage in *Gsn*^*-/-*^ platelets that was largely insignificant. Consequently, it does not appear that disruptions of the cytoskeleton (and transient effects on PS exposure) have a significant bearing on platelet survival. These observations invite the speculation that the cytoskeleton may regulate specific spatial/temporal aspects of ABT-737-driven PS exposure.

In conclusion, the present study demonstrates that ABT-737-induced platelet apoptosis occurs independently of the actin cytoskeleton. The platelet’s anucleate nature and unique structural characteristics likely contribute to a distinct programmed death response following exposure to pro-apoptotic stimuli. It is therefore of considerable interest to identify the exact determinants of the pertinent signaling pathways to identify potential therapeutic targets for conditions characterized by thrombocytopenia.

## Supporting information

S1 Data (ZIP)

S1 Raw images (PDF)
