# Supplementary material for: Apoptosis in platelets is independent of the actin cytoskeleton
Source: PLoS One. 2022 Nov 15;17(11):e0276584. doi: 10.1371/journal.pone.0276584 (PMC9665360; doi:10.1371/journal.pone.0276584)
Supplement: S1 Raw images — (PDF) [file pone.0276584.s002.pdf]

raw_images_for_figure_2A

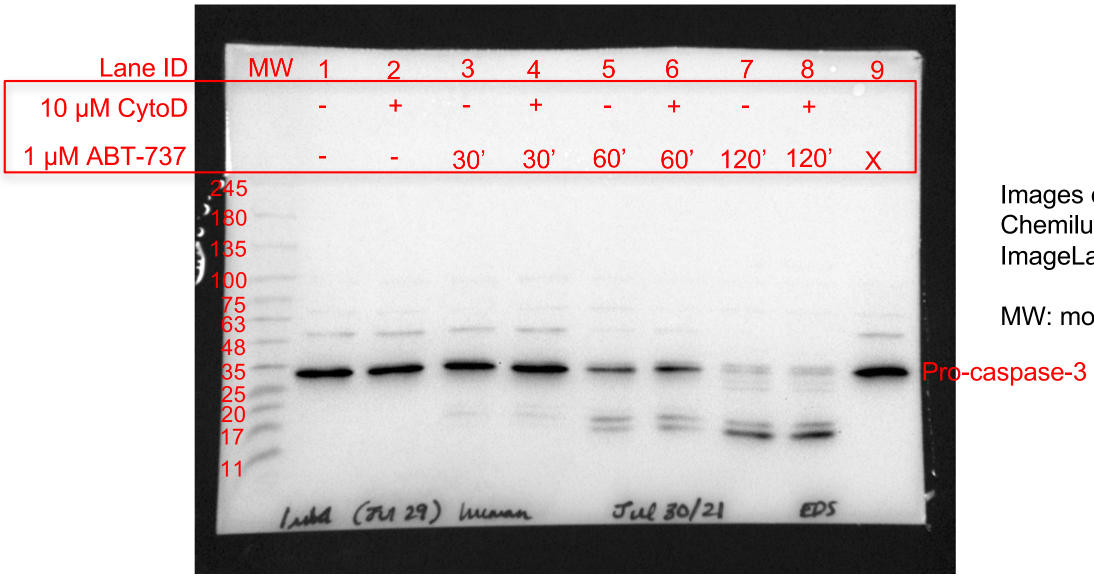

Images captured by Chemiluminescent Hi-Res on ImageLab Software

MW: molecular weight marker

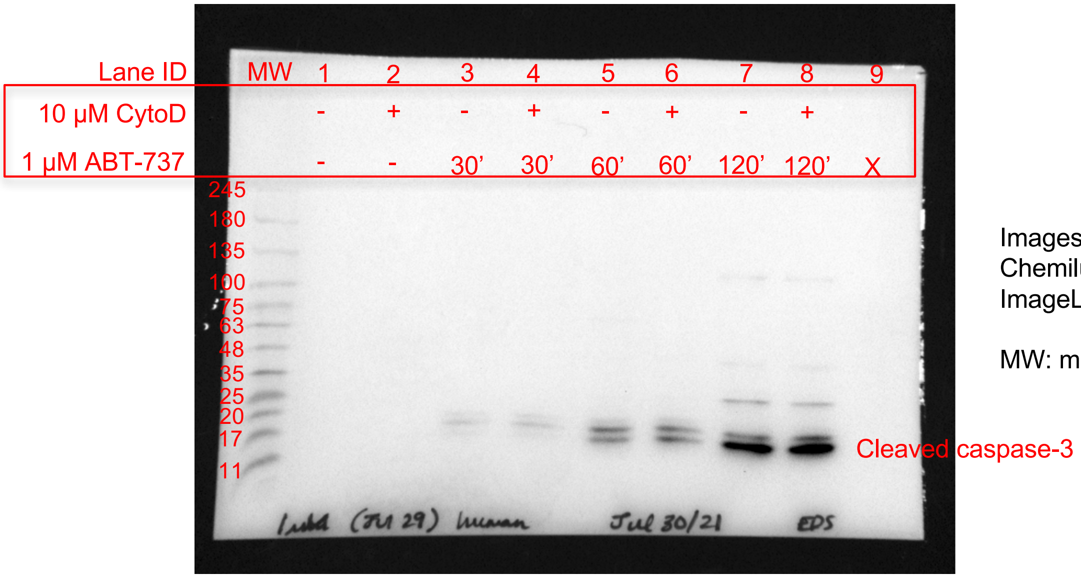

Images captured by Chemiluminescent Hi-Res on ImageLab Software

MW: molecular weight marker

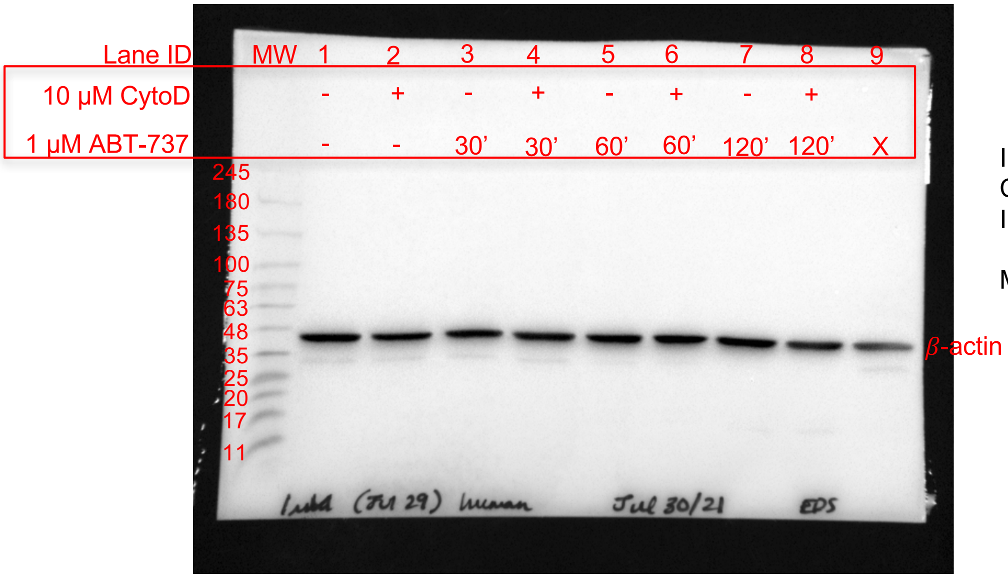

Images captured by Chemiluminescent Hi-Res on ImageLab Software

MW: molecular weight marker

raw_images_for_figure_2C

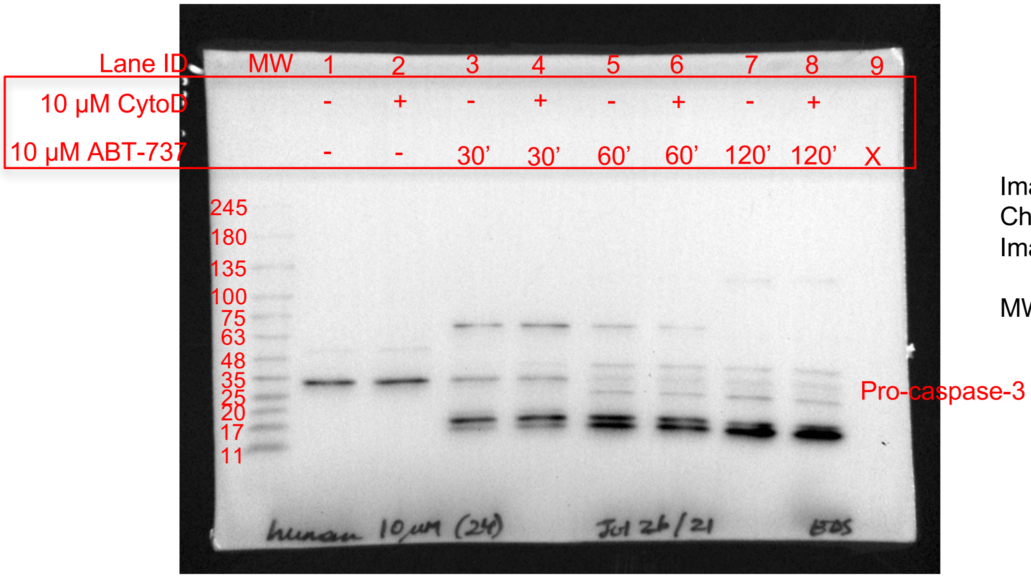

Images captured by Chemiluminescent Hi-Res on ImageLab Software

MW: molecular weight marker

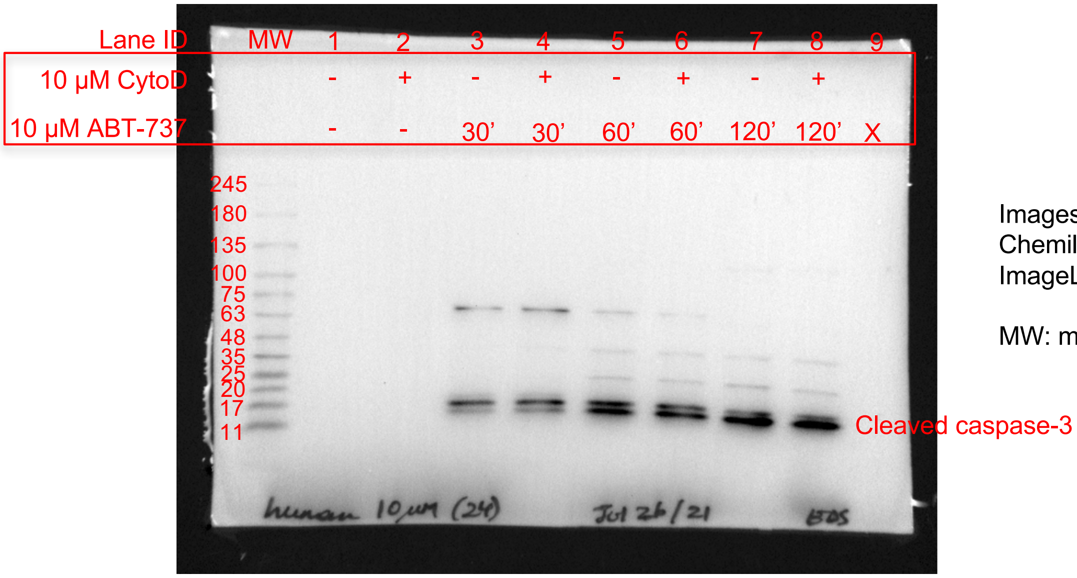

Images captured by Chemiluminescent Hi-Res on ImageLab Software

MW: molecular weight marker

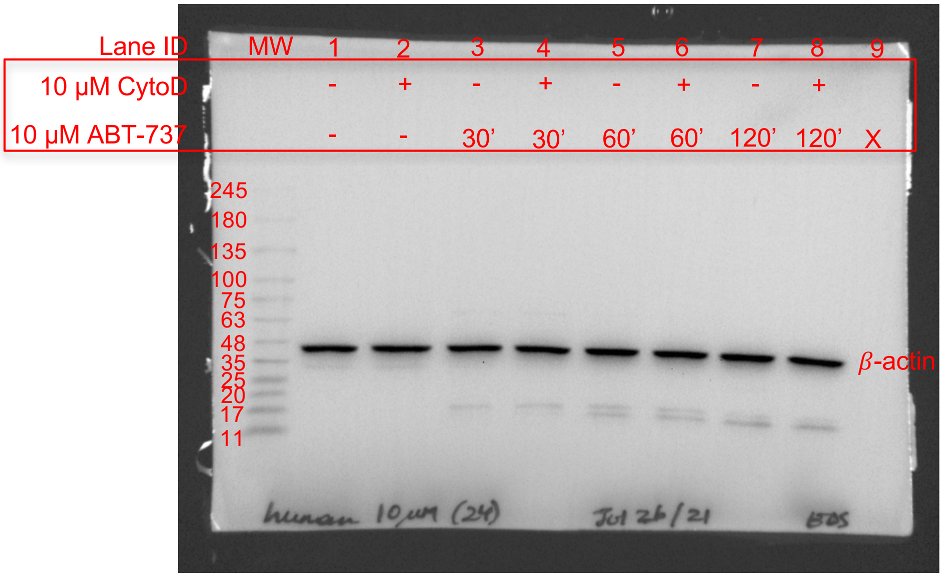

Images captured by Chemiluminescent Hi-Res on ImageLab Software

MW: molecular weight marker

raw_images_for_figure_4A

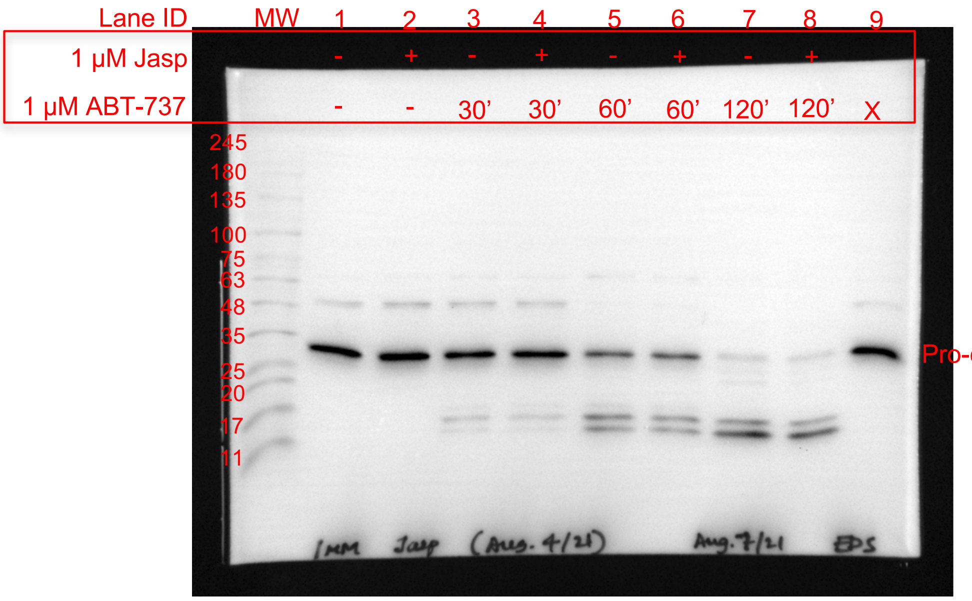

Images captured by Chemiluminescent Hi-Res on ImageLab Software

MW: molecular weight marker

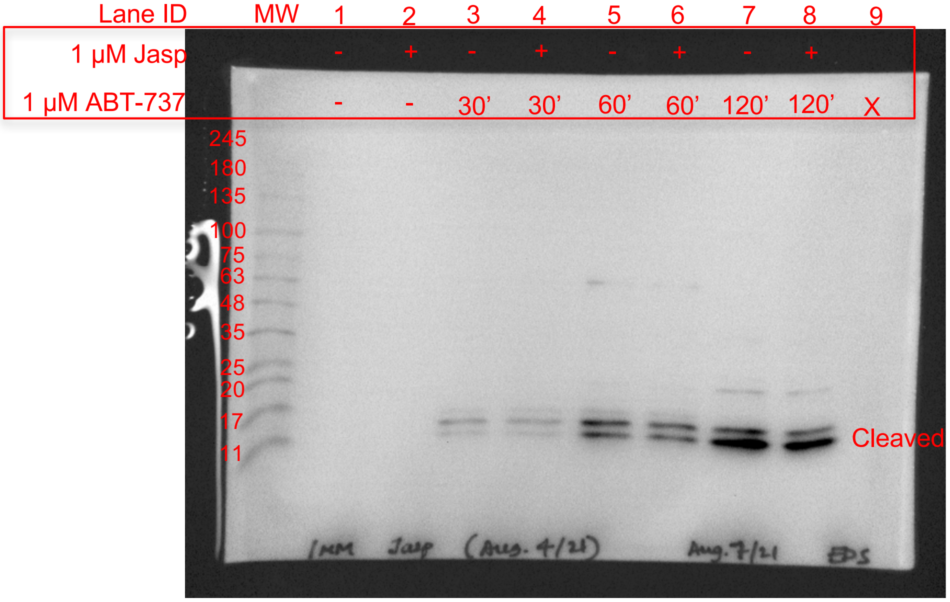

Images captured by Chemiluminescent Hi-Res on ImageLab Software

MW: molecular weight marker

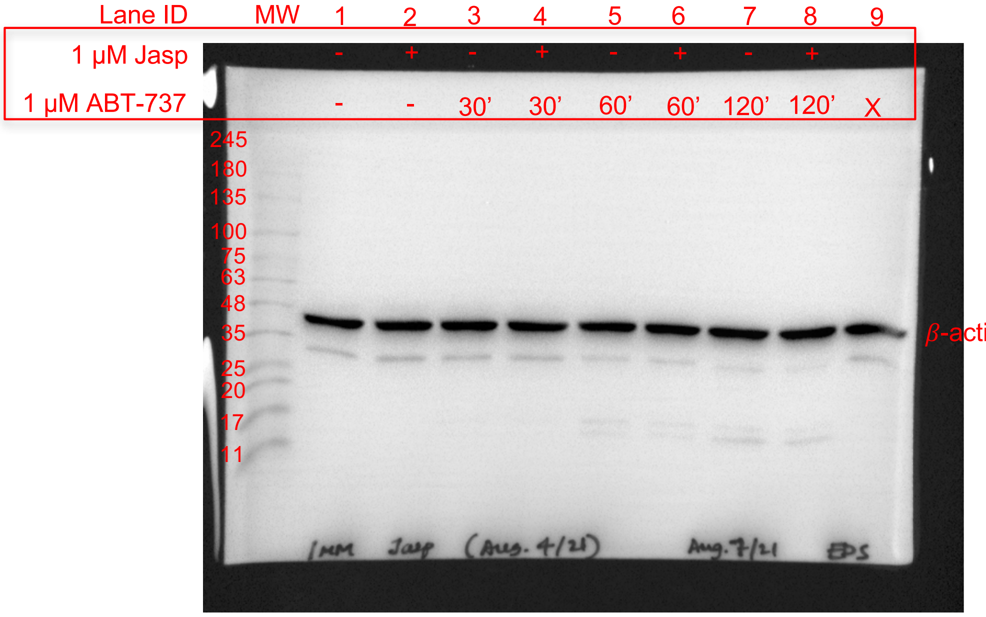

Images captured by Chemiluminescent Hi-Res on ImageLab Software

MW: molecular weight marker

raw_images_for_figure_4C

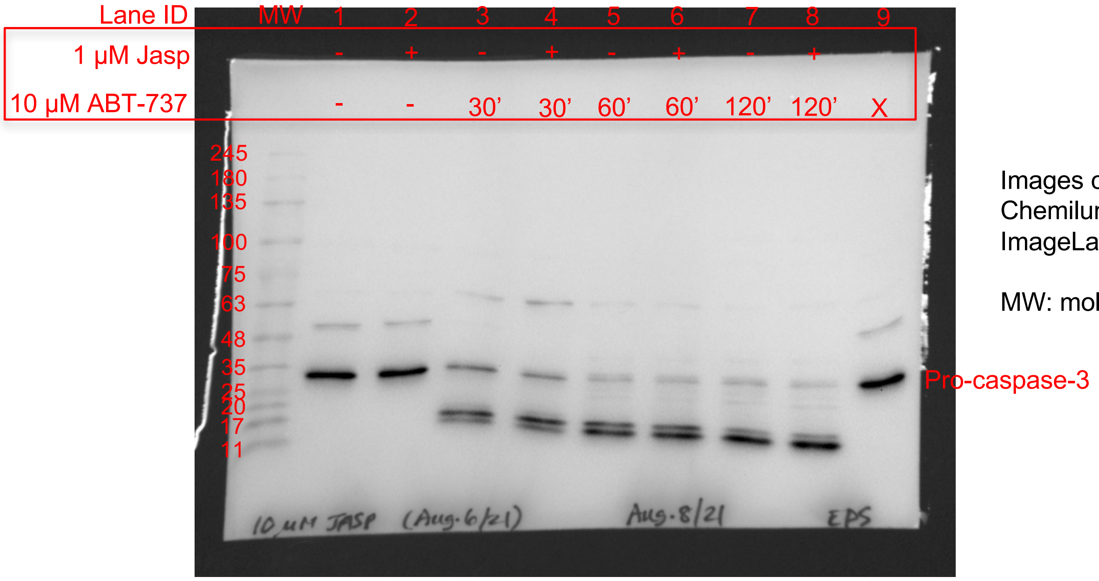

Images captured by Chemiluminescent Hi-Res on ImageLab Software

MW: molecular weight marker

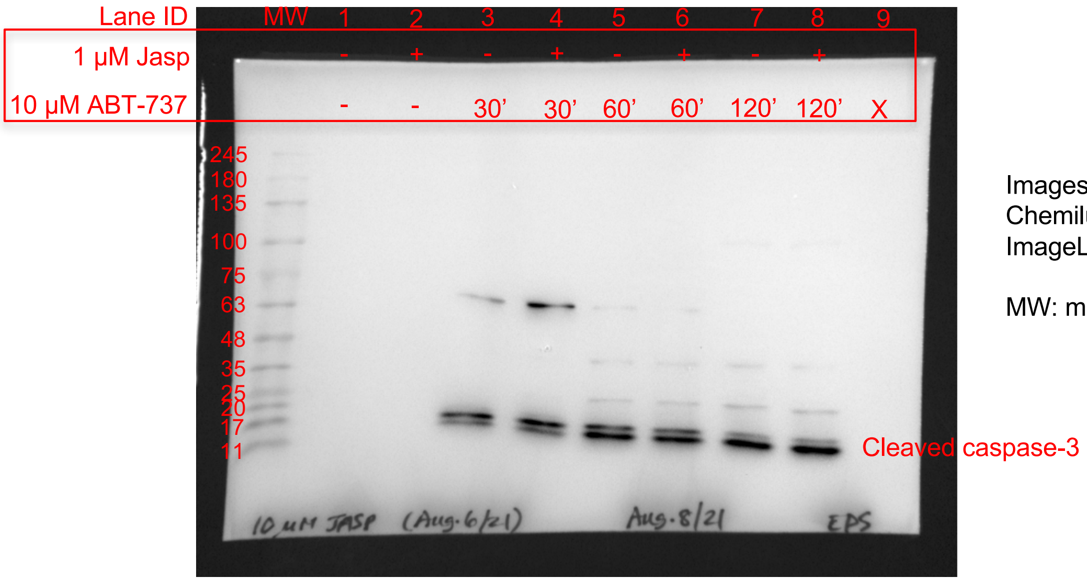

Images captured by Chemiluminescent Hi-Res on ImageLab Software

MW: molecular weight marker

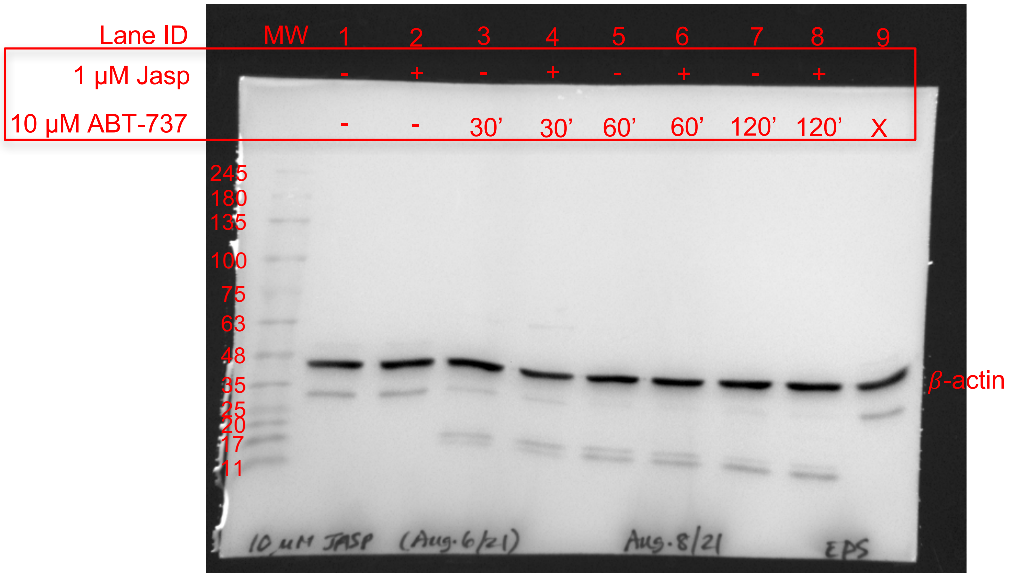

Images captured by Chemiluminescent Hi-Res on ImageLab Software

MW: molecular weight marker

raw_images_for_figure_5A

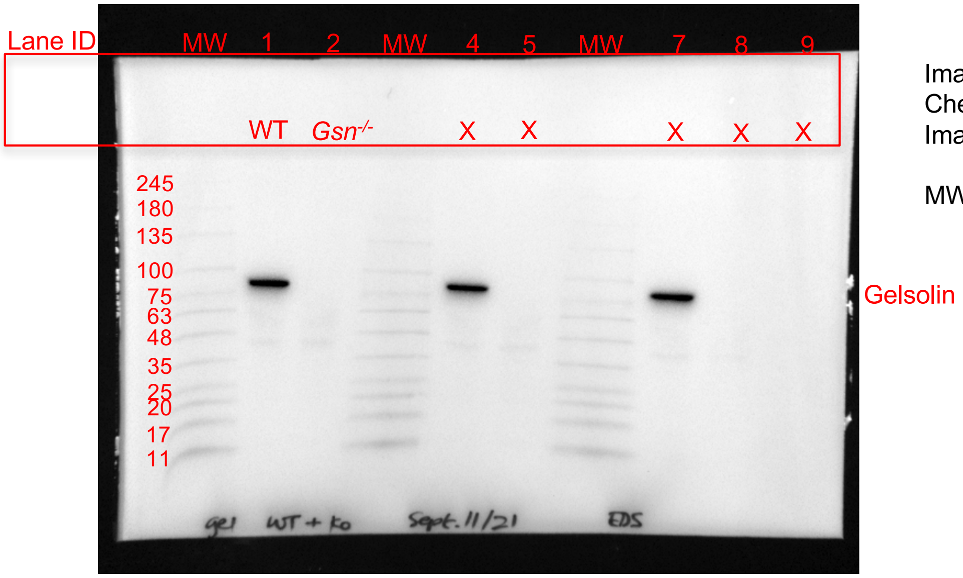

Images captured by Chemiluminescent Hi-Res on ImageLab Software

MW: molecular weight marker

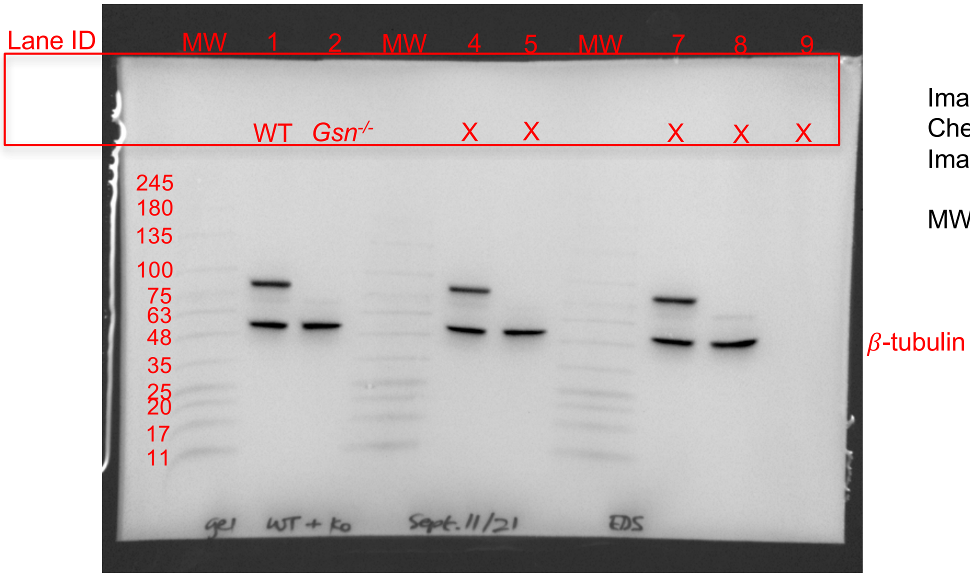

Images captured by Chemiluminescent Hi-Res on ImageLab Software

MW: molecular weight marker

raw_images_for_figure_6A

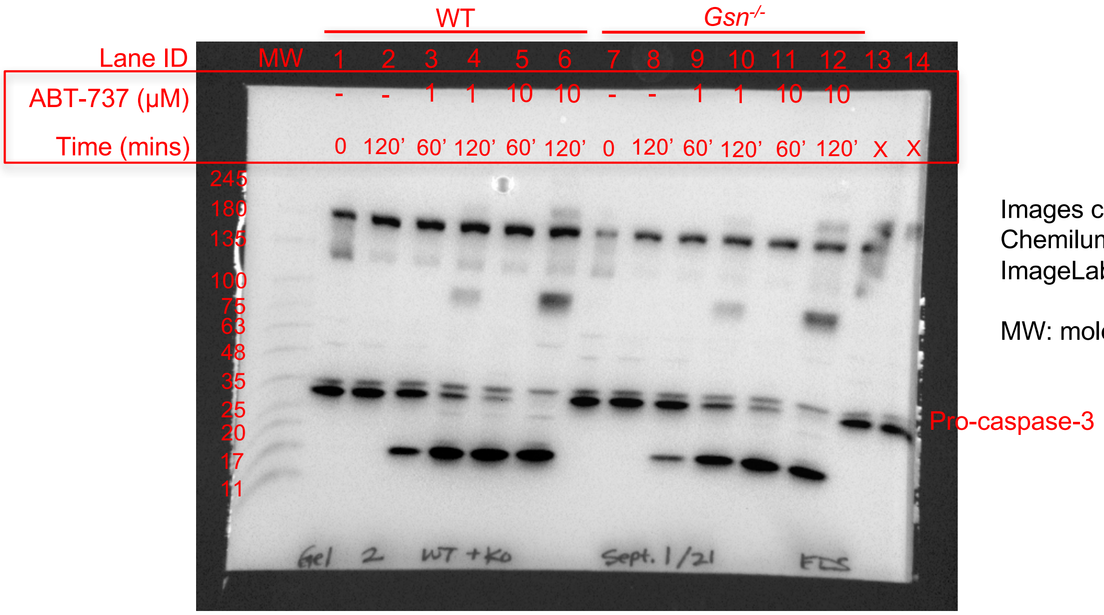

Images captured by Chemiluminescent Hi-Res on ImageLab Software

MW: molecular weight marker

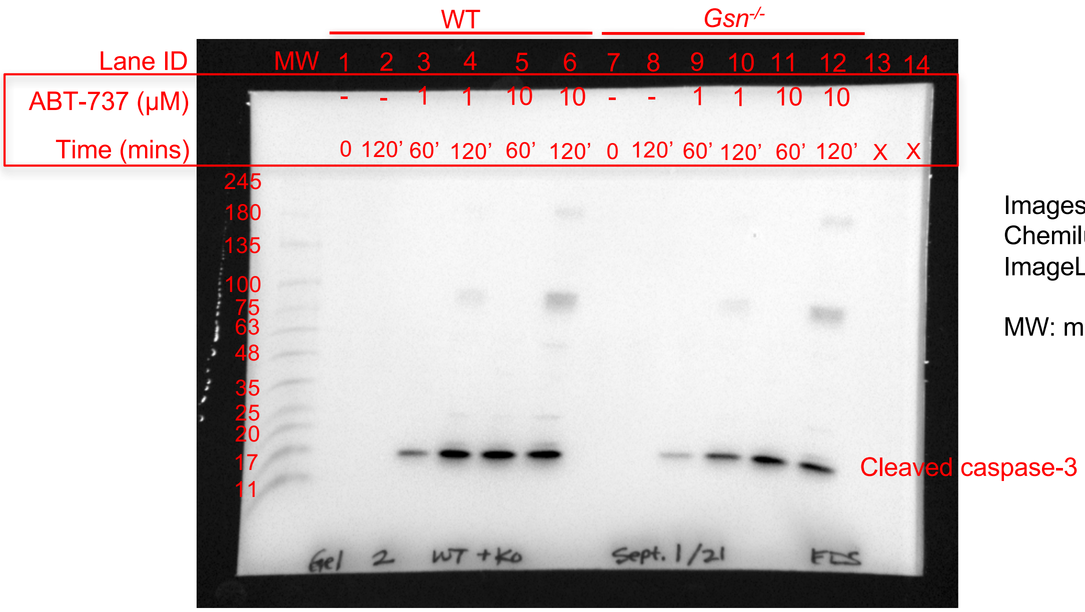

Images captured by Chemiluminescent Hi-Res on ImageLab Software

MW: molecular weight marker

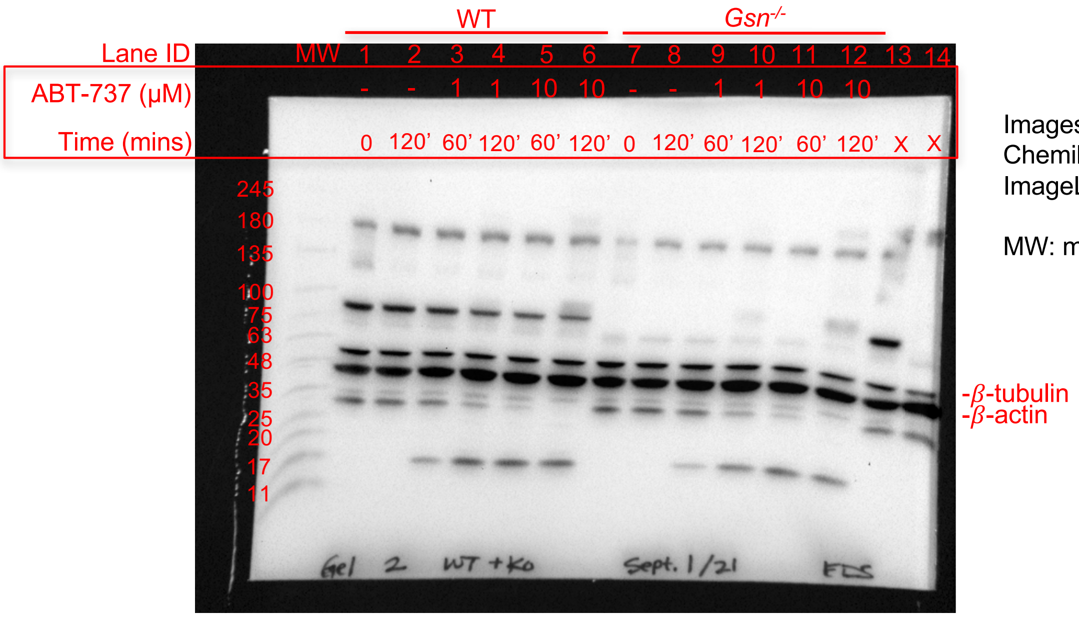

Images captured by Chemiluminescent Hi-Res on ImageLab Software

MW: molecular weight marker
